# Impact of drug-resistant tuberculosis on socio-economic status, quality of life and psychological well-being of patients in Bucharest, Romania: a prospective cohort study

**DOI:** 10.1186/s41043-024-00717-x

**Published:** 2024-12-22

**Authors:** Rupa Ramachandran, Andreea Dumitrescu, Dragos Baiceanu, Cristina Popa, Antonela Dragomir, Beatrice Mahler, Michael Hoelscher, Christoph Lange, Jan Heyckendorf, Andrea Rachow, Elmira Ibraim, Olena Ivanova

**Affiliations:** 1 Deggendorf Institute of Technology, European Campus Rottal-Inn, 84347 Pfarrkirchen, Germany; 2 Marius Nasta Institute of Pneumology - German Centre for Infection Research (DZIF), Eastern European Study Site, Bucharest, Romania; 3 Carol Davila University of Medicine and Pharmacy, Bucharest, Romania; 4 Institute of Infectious Diseases and Tropical Medicine, LMU University Hospital, LMU Munich, Munich, Germany; 5 German Centre for Infection Research (DZIF), Partner Site Munich, Munich, Germany; 6 Unit Global Health, Helmholtz Zentrum München, German Research Center for Environmental Health (HMGU), Neuherberg, Germany; 7 Fraunhofer Institute for Translational Medicine and Pharmacology ITMP; Immunology, Infection and Pandemic Research, Munich, Germany; 8 Division of Clinical Infectious Diseases, Research Center Borstel, Borstel, Germany; 9 German Centre for Infection Research (DZIF), partner site Hamburg-Lübeck-Borstel-Riems, Borstel, Germany; 10 Respiratory Medicine and International Health, University of Lübeck, Lübeck, Germany; 11 Baylor College of Medicine and Texas Children’s Hospital, Houston, TX USA; 12 Department of Internal Medicine I, Kiel University and University Medical Center Schleswig-Holstein, Campus Kiel, Kiel, Germany

**Keywords:** Tuberculosis, Drug-resistant, Quality of life, Psychological distress, Socio-economic impact

## Abstract

**Background:**

Tuberculosis (TB) remains a global health challenge, with 1.3 million deaths in 2022. Ten countries in the European Union (EU) and European Economic Area (EEA) accounted for 88.3% of TB cases, of which 23.8% were from Romania. Evidence shows that mental health issues, decreased quality of life and negative socio-economic impact are common among TB patients; however, there is limited evidence available in Romania. The main aim of this study is to longitudinally assess the quality of life, mental health, and socio-economic status of patients with drug-resistant TB (DR TB) in Romania.

**Methods:**

A prospective cohort study was conducted at the Marius Nasta Institute of Pneumology in Bucharest, Romania, enrolling 50 participants with DR TB. Demographic data, clinical examinations, laboratory test and medical history were recorded at study start. At baseline (week two), month 10, and month 20 we also administered the Short-Form-36 and Kessler Psychological Distress Scale to assess health-related quality of life and mental health status, and socio-economic questionnaires to 46 participants.

**Results:**

Of the 46 participants with median age of 48.9 years, 71.7% were males. Majority of the participants were employed at the baseline but due to the rigorous treatment and hospitalization had to take sick leave, thereby affecting the individual and household income. 26.1% and 39.3% of participants reported psychological distress at baseline and at the end of month 20, respectively. The quality-of-life scores improved during treatment: PCS with a mean of 67.0 (SD-33.9) at baseline, 63.3 (SD-31.9) at month 10 and 70.3 (SD-30.3) at month 20, and MCS with 62.8 (SD-30.6), 67.8 (SD-29) and 70.8 (SD-27.3), accordingly, but differences were not significant.

**Conclusions:**

We examined the socio-economic impact, quality of life, and psychological distress among patients affected by DR TB in Romania. The results of this study suggest that social and psychological support will ensure a better standard of living during and following TB treatment.

**Supplementary Information:**

The online version contains supplementary material available at 10.1186/s41043-024-00717-x.

## Background

Tuberculosis (TB) is one of the leading causes of death worldwide, claiming the lives of 1.3 million people in the year 2022 [1]. Globally it is estimated that 10.6 million people are affected by TB and over 80% of the cases are in low- and middle-income countries [1]. Thirty countries belonging to the European Union (EU) and European Economic area countries (EEA) reported 33,527 TB cases in the year 2021. The notification rate of TB was 7.4 per 100,000 population in the EU/EEA countries [2]. The recent TB surveillance report by World Health Organisation (WHO) has stated an increase in the incidence of TB by 1.2% in the WHO European region in the year 2021 [3]. The report also indicates the rise of multidrug-resistant TB (MDR-TB), or of rifampicin-resistant TB (RR-TB) as a surrogate for MDR-TB, in this region with estimated new cases of 72,000 [3]. In 2021, ten countries in the EU/EEA accounted for 88.3% and Romania alone accounted for 23.8% of the total TB cases [2]. A total of 7,979 TB cases were documented in Romania, corresponding to a notification rate of 41.6 per 100,000 population. This notification rate is five times higher than EU/EEA average [2]. Romania also accounted for 24 out of the 115 pre-XDR cases reported in the EU/EEA [3]. Furthermore, out of 758 DR TB cases that were reported in the EU/EEA, 264 were from Romania [4].

Globally, evidence suggests that TB impacts mental health and quality of life of patients. The prevalence of depression and depressive symptoms among TB patients in the WHO European Region has been found to be between 19.0% and 65.0% [5]. In Romania, the prevalence of depression among the hospitalised TB patients was between 38.9% and 65.0% and depressive symptoms were higher among patients with DR TB [5]. TB coupled with depression can worsen the burden of the disease and can have a detrimental impact on the quality of life [5].

Health-related quality of life (HRQoL) is a holistic concept encompassing various aspects of an individual’s well-being, such as physical, mental, emotional, and social functioning. Unlike solely considering life expectancy and causes of death, HRQoL emphasizes the influence that health status has on an individual’s overall quality of life [6]. Several studies have found that TB affects the quality of life, thereby rendering the patients being psycho-socially weak [7–10]. In terms of socio-demographic factors, age and gender were linked to worse QoL scores. Illiteracy and low socio-economic status had deleterious effect on the mental health domain of QoL [7, 10]. Improvements in the QoL scores were seen during TB treatment, but there was a compromise in the scores at the initiation and completion of the treatment [7].

Moreover, in addition to negative health outcomes, TB impacts the financial situation of patients and their households. A recent meta-analysis by Portnoy et al. demonstrated that 54.9% TB-affected households experience catastrophic costs due to TB diagnosis and treatment, with poorer households more likely to experience catastrophic expenditure than richer ones [11]. Thus, despite the availability of free diagnosis and treatment for TB, there are substantial costs incurred by patients and their families.

Despite the global evidence, there is a limited amount of literature analysing the change in mental health status, quality of life and socio-economic status from diagnosis to the end of the treatment in Romania. Most of the literature explores the burden of TB and its clinical manifestations in general population as well as vulnerable groups in Romania [12, 13]. Hence, this study aims to fill existing gap and explore the multifaceted impact of DR TB on patients in Romania including mental health, quality of life and socio-economic impact.

## Methods

### Study design

Prospective cohort study design was employed to assess clinical outcomes of TB and the changes in socio-economic indicators, quality of life and mental health domain throughout the treatment phase.

### Study setting

Romania has a population of 19.32 million and is one among the six countries in the EU with high burden of TB of 8,700 cases in 2021 [3]. This study was conducted at the Marius Nasta Institute of Pneumology in Bucharest, which hosts the National Tuberculosis Program (NTP) and the National TB Reference Laboratory. It is also the Eastern European Study Site of the German Center for Infection Research (DZIF).

### Data collection

Patients with acid fast bacilli sputum smear positive TB, confirmed by the nucleic acid amplification testing (GeneXpert), and who agreed voluntarily to be part of the study were included. Patients who were undergoing anti-TB therapy within the last 6 months; diagnosed with HIV infection; relevant immunosuppression at the physician’s discretion; <18 years of age; in custodianship or guardianship; anticipated inability to follow the study requirements, to finish the study due to physical weakness or anticipated migration were excluded from the study.

Prior to the initiation of treatment, demographic data, TB diagnostic tests data, medical history, TB symptoms, chest X-Rays and information regarding environmental and behavioural risk factors were collected. Data on TB diagnostic tests, TB treatment and adherence were assessed at regular intervals from anti-TB treatment start: two weeks, month 2, 4, 6, 10, 15 and 20. Data regarding environmental and behavioural risk factors were obtained during the follow-up period at 10th and 20th months. Information on socio-economic variables, QoL and mental health was collected at two weeks, month 10 and 20.

SF-36 was used to assess QoL, which is a patient-reported multiple-choice questionnaire with 36 questions covering eight domains such as physical functioning, role physical, bodily pain, general health, vitality, social functioning, role emotional, and mental health. The responses were scored, and a mental and physical component summary (MCS and PCS) was obtained [8]. Physical functioning reflects a person’s ability to carry out day-to-day activities and role functioning domains defines the person’s ability to carry out the specified role at a specific place such as work, home etc. The social functioning touches the topics such as stigmatization, losing close relations and friends and social isolation. After receiving a TB diagnosis, the psychological mindset of the patients is altered. The psychological and emotional domain measures these alterations [7].

Kessler Psychological distress scale (K-10) is a symptom-oriented questionnaire measuring global psychological distress for a period of 30 days. K-10 scale comprises of 10 questions, each with a response ranging from “Not at all” to “All the time”. The scale helps in identifying people with not only clinically defined depression or anxiety disorders but also identify people with sub-clinical illness which doesn’t formally satisfy clinical definitions for specific conditions [14]. Patients with scores less than 20 were deemed as mentally fit, between 20 and 24 as having a mild psychological distress, 25–29 as moderate psychological distress and more than 30 as severe psychological distress.

The socio-economic burden of TB was assessed using an adapted tool from TB Sequel study [15]. The questionnaire used in the study was a modification of the WHO generic TB patient cost instrument and the adapted version can be used to compare the socio-economic changes over time [16]. The data collected in this study includes information on coping mechanisms and socio-economic status at week two, months 10 and months 20.

In the current study, a study identification code had been attributed to each participant at enrolment and all individual data and information have been collected on paper forms, uploaded into the electronic database and analysed anonymously.

### Data analysis

The statistical analysis was performed using the R software version 4.2.1. The categorical variables were summarized by frequency tables and continuous variables as measures of central tendency. The responses to the QoL questionnaire and K-10 questionnaire were scored according to the SF-36 and K-10 scoring manuals. The difference between the baseline and follow-up scores were assessed using the analysis of variance test (ANOVA) for both QoL scores and K-10 scores. ANOVA test was used again to measure the difference in the 20th month QoL scores based on sex, age and history of TB and chi-square test was used to measure the difference in K-10 scores based on age, sex, history of TB, marital status, and employment status. The significance of p-value was set as < 0.05. For the SF-36 QoL scores, alpha Cronbach values were calculated.

## Results

### Demographic and TB characteristics

**Fig. 1:**
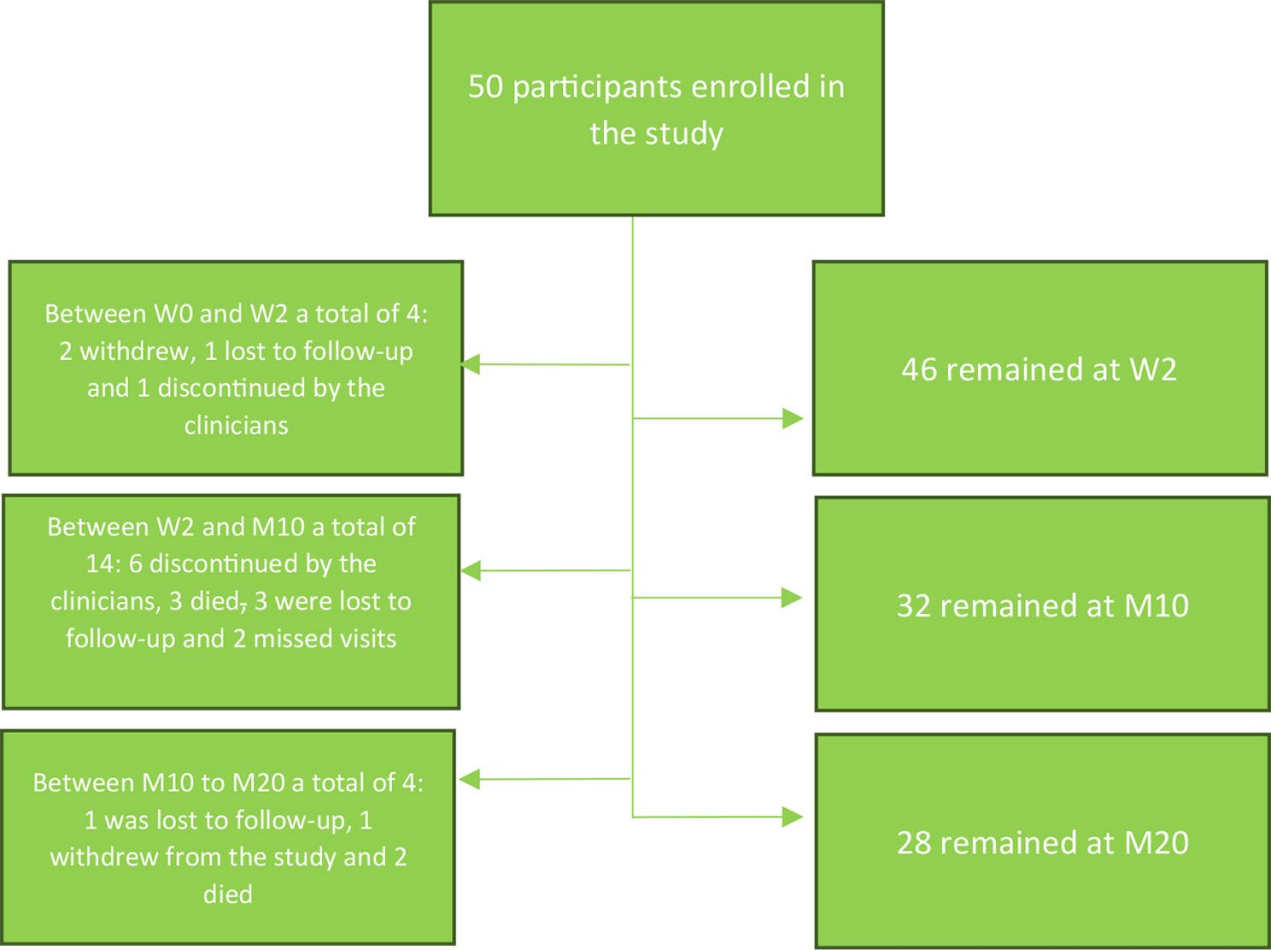
Study flow diagram

Fifty subjects diagnosed with DR TB were enrolled from May 2019 to July 2021. During the 10 months follow-up, 18 participants withdrew from the study due to reasons cited below. At month 20, 28 remained in the study (Fig. 1).

Of the 50 participants enrolled in the study, we present the socio-demographic characteristics of 46 who filled in SF36, QoL and K10 questionnaires at week two after enrolment (“baseline”). A large proportion of the study population (71.7%) were males. Majority of the participants (47.8%) were aged 50 years and above and the median age at baseline was 48.9 years. 34.8% of the participants had tertiary or higher education, 32.6% had vocational training, and 65.2% were employed. More than half of the participants (63.04%) were either married or living with a partner. A total of 48.0% of the participants had a previous history of TB and the most common symptom reported was cough (95.7%) followed by weight loss (71.7%) and night sweats (50.0%). The median BMI was found to be 19.7 with an interquartile range between 16.6 and 22.5. Many of the participants were cigarette smokers (58.7%) and 34.2% consumed alcohol 4 or more times per week. Hypertension was found to be a major comorbidity among the participants. The variable age, sex, marital status, employment status, education and previous history of TB were compared between the group that completed the study and the group that discontinued from the study with no significant differences found (see Annex 1). A detailed description of baseline socio-demographic characteristics and medical history are presented in Table 1.

**Table 1:** Baseline socio-demographic characteristics and medical history of DR TB participants (*N* = 46)

Characteristics		
Sex		% (n/N)
	Male Female	71.7% (33/46) 28.3% (13/46)
Age		
	Median (SD) < 30 yrs 30–39 40–49 >/=50	48.9 (15.31) 13.0% (6/46) 15.2% (7/46) 23.9% (11/46) 47.8% (22/46)
Education		
	Primary Secondary High school Vocational training University and higher	10.9% (5/46) 21.7% (10/46) 28.3% (13/46) 32.6% (15/46) 6.5% (3/46)
Marital status		
	Single Married/Living with partner Divorced/Separated/ Widowed	21.7% (10/46) 63.0% (29/46) 15.2% (7/46)
Employment ^a^		
	Employed (Technician, Government office, Factory worker, Service) Unemployed (Retiree, Student, Homemaker, Unemployed)	65.2% (30/46) 32.6% (15/46)
Number of children		
	None 1–2 3–4 5–7	21.7% (10/46) 54.4% (25/46) 21.7% (10/46) 2.2% (1/46)
Dependents apart from children		
	Yes No	10.9% (5/46) 89.1% (41/46)
Primary income		
	Patient Wife/Mother Husband/Father Son/Daughter Other	67.4% (31/46) 6.5% (3/46) 13.0% (6/46) 4.4% (2/46) 8.7% (4/46)
Main occupation ^a^		
	Service Retiree Factory worker Technician Unemployed Homemaker Student Government employee	43.5% (20/46) 17.4% (8/46) 13.0% (6/46) 6.5% (3/46) 6.5% (3/46) 4.4% (2/46) 4.4% (2/46) 2.2% (1/46)
History of TB		
	Yes No	48.0% (21/46) 52.0% (25/46)
TB symptoms		
	Cough	95.7% (44/46)
	Weight loss	71.7% (33/46)
	Night sweats	50.0% (23/46)
	Bloody cough	21.7% (10/46)
	Fever	15.2% (7/46)
BMI		
	Median (IQR)	19.7 (16.64–22.50)
Smoking ^a^		
	Currently smoking	58.7% (27/46)
	Past smoking	32.6% (15/46)
Alcohol consumption		
	Yes No	82.6% (38/46) 17.4% (8/46)
Frequency of alcohol consumption		
	Monthly or less 2–3 times a month 2–3 times a week 4 or more times a week	29.3% (12/38) 17.1% (5/38) 19.5% (7/38) 34.2% (14/38)
Drugs ever use		
	None	100% (46/46)
Imprisoned		
	Yes No	10.9% (5/46) 89.1% (41/46)
Work in mines		
	Yes No	8.7% (4/46) 91.3% (42/46)
Comorbidities*		
	Diabetes	2.2% (1/46)
	Hypertension	15.2% (7/46)

*Two patients reported allergies, one reported chronic hepatitis, three reported COPD, one reported cancer, one reported visual impairment, one reported renal issue, two reported neurological disorder and three reported psychological disorder. Patients were asked about asthma, silicosis, sarcoidosis, heart failure, renal failure, STIs, with no patients indicating that they had current issues with the previously stated comorbidities

^a^ Missing observation: Employment for 1 participant, main occupation for 1 participant, past smoking for 20 participants

### Socio-economic impact of TB

At the treatment start, 17.4% of the participants had to use their household savings for managing their treatment costs and 4.4% had borrowed money. Initially only 8.7% of the participants received a social welfare payment but at the end of the month 20, it increased to 60.7%. Similar trend was observed in receiving vouchers or goods. When questioned about the impact of TB, 60.9% had responded that TB affected their social and private life. At the month 10 and 20, 18.8% and 14.3% respectively stated that they had very serious financial impact of TB (Table 2).

**Table 2:** Socio-economic impact of TB

Outcome/characteristic	Baseline (*N* = 46)	M10 (*N* = 32)	M20 (*N* = 28)
**Coping costs - use of household savings**			
(Yes, n/N)	17.4% (8/46)	21.9% (7/32)	14.3% (4/28)
(No, n/N)	82.2% (38/46)	78.1% (25/32)	85.7% (24/28)
**Borrowed money to cover the cost of illness**			
(Yes, n/N)	4.4% (2/46)	18.8% (6/32)	17.9% (5/28)
(No, n/N)	95.7% (44/46)	81.3% (26/32)	82.1% (23/28)
**Financial impact of TB on household**			
No impact	45.7% (21/46)	28.1% (9/32)	39.3% (11/28)
Little impact	21.7% (10/46)	21.9% (7/32)	25.0% (7/28)
Moderate impact	17.4% (8/46)	25.0% (8/32)	21.4% (6/28)
Serious impact	6.5% (3/46)	6.3% (2/32)	0%
Very serious impact	8.7% (4/46)	18.8% (6/32)	14.3% (4/28)
**Social welfare payment**			
(Yes, n/N)	8.7% (4/46)	59.4% (19/32)	60.7% (17/28)
(No, n/N)	91.3% (42/46)	40.6% (13/32)	39.3% (11/28)
**Received vouchers or goods**			
(Yes, n/N)	2.2% (1/46)	40.6% (13/32)	50.0% (14/28)
(No, n/N)	97.8% (45/46)	59.4% (19/32)	50.0% (14/28)
**Has TB affected social and private life**			
(Yes, n/N)	60.9% (28/46)	62.5% (20/32)	64.3% (18/28)
(No, n/N)	39.1% (18/46)	37.5% (12/32)	35.7% (10/28)

### Employment and income trend

**Fig. 2:**
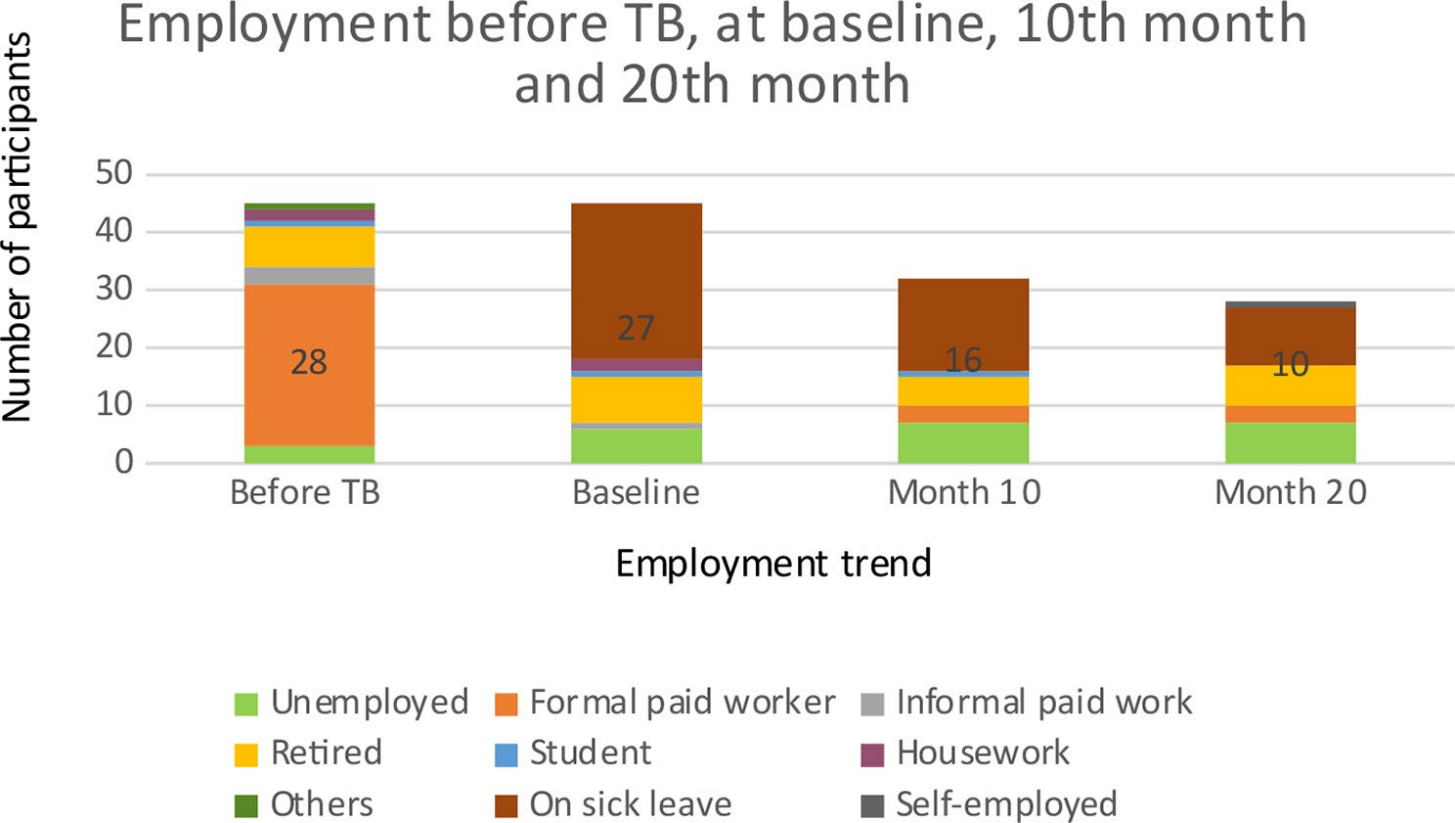
Employment trend - before TB, at the baseline, month 10, and month 20

Before the TB diagnosis, more than half of the participants (60.9%) were employed as formal paid worker and 15.2% had retired. At the baseline, at month 10 and months 20, the employment status of the majority shifted from being employed to being on sick leave. More than half (58.7%) were on sick leave at baseline, 50.0% at month 10 and 35.7% at month 20. Unemployment also increased during the duration of the treatment. At the baseline, only 3 (6.5%) participants were unemployed but at the end of month 20, 7 (25.0%) participants remained unemployed (Fig. 2). A total of 31 participants stated that they had to stop working due to TB-related illness.

### Personal income

**Fig. 3:**
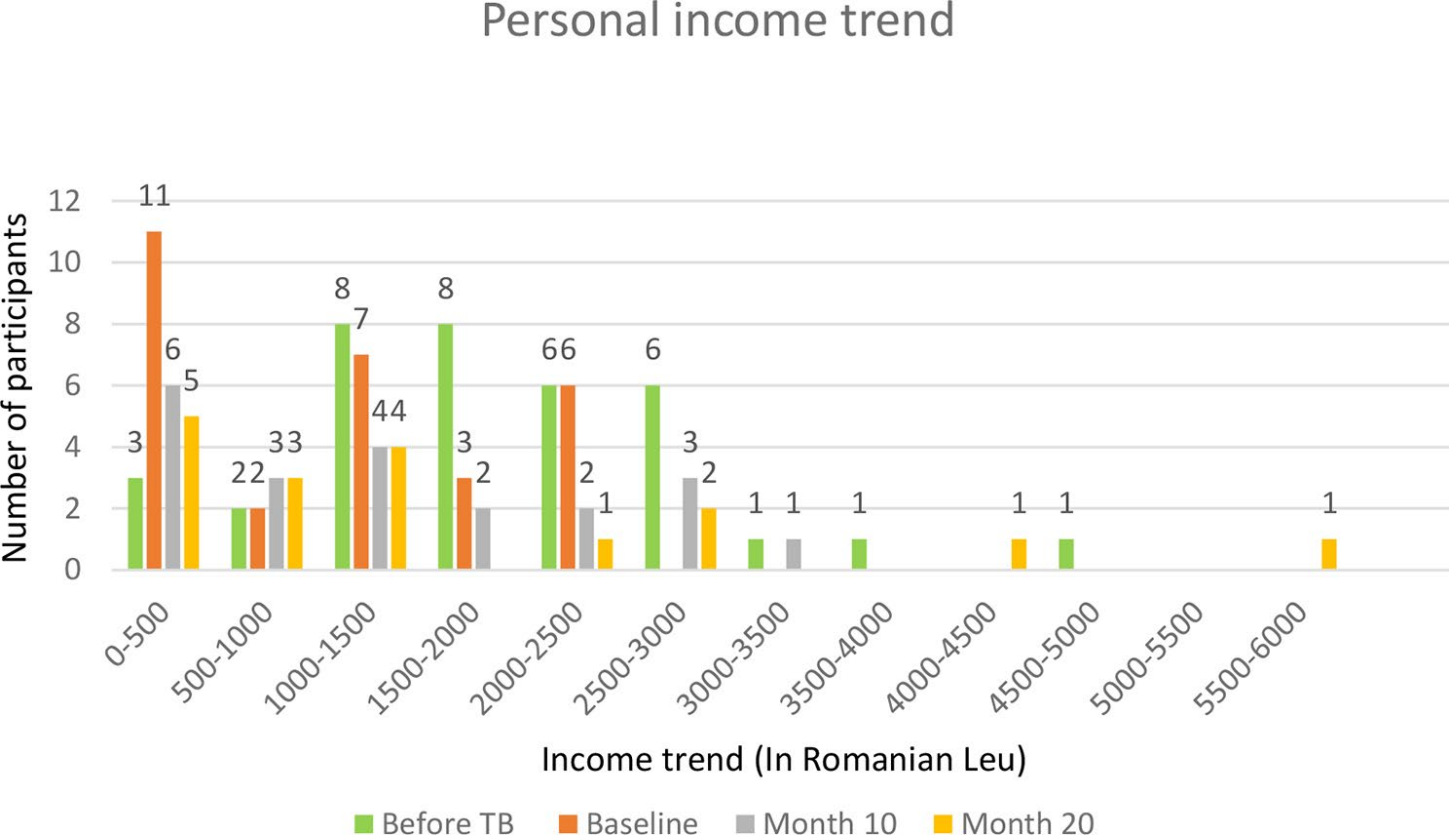
Personal income trend – before TB, at the baseline, at month 10 and month 20

A total of 36 participants had mentioned their personal income before the diagnosis of TB and 29 mentioned their personal income at baseline. Not all the participants revealed their personal and family income. Before the TB diagnosis, 16 (34.8%) participants had an income between 1,000 RON and 2,000 RON and 12 (26.1%) had an income range between 2,000 RON and 3,000 RON (1 EUR is approximately 5 RON and the net minimum salary in Romania in 2023 was set at 1863 RON (373.90€)). This can be attributed to the fact that majority of them were employed in the formal sector. At baseline, only 7 (15.2%) reported their income range to be between 1,000 and 1,500 RON and 11 (23.9%) of them had an income range of 0-500 RON. The change in the income could be due to the change in employment status from employed to being on sick leave. At month 10, 6 (18.8%) of them received an income between 0 and 500 RON and 8 (25.0%) had an income between 1,000 RON and 2,500 RON. At month 20, 3 (10.7%) had an income between 500 RON and 1,000 RON, and 5 (17.8%) had income range between 0 RON and 500 RON. See Fig. 3 for more details.

### Household income

**Fig. 4:**
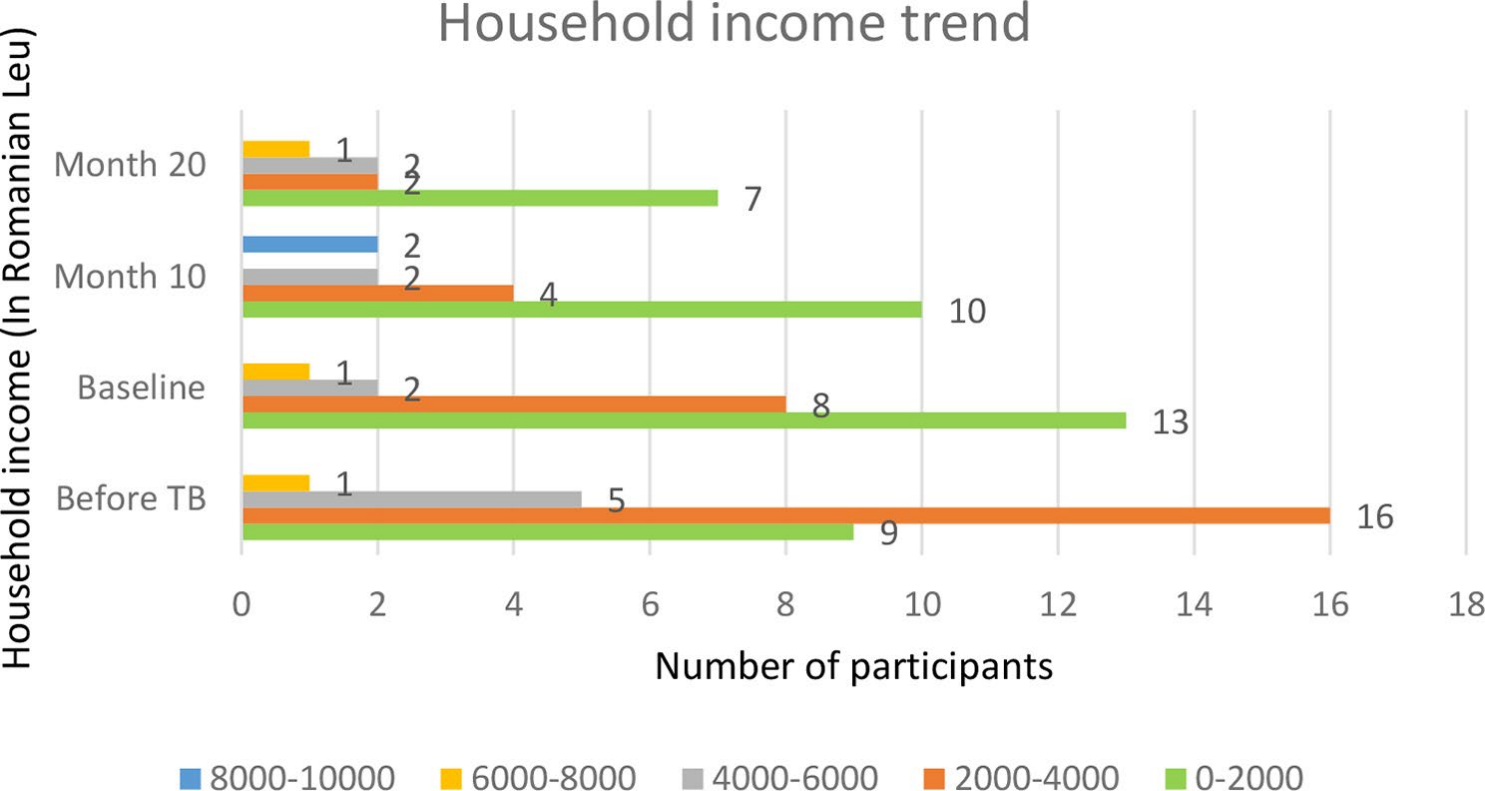
Household income trend – before TB, at the baseline, at month 10 and month 20

Before TB diagnosis, 16 (34.8%) of the participants had a household income between 2,000 RON and 4,000 RON. However, at baseline, month 10 and month 20 the majority of the participant’s household income declined to the range between 0 RON and 2,000 RON (Fig. 4).

### Quality of life over time

Participants reported a physical health component summary score (PCS) with a mean of 67.0 (SD 33.9) at baseline, 63.3 (SD 31.9) at month 10 and 70.3 (SD 30.3) at month 20. The mean mental health component summary score (MCS) was 62.8 (SD 30.6) at baseline, 67.8 (SD 29.0) at month 10 and 70.8 (SD 27.3) at month 20. Although the scores in all the domains except role physical and bodily pain seemed to be improving differences were not significant. Table 3 displays the scores in all the domains obtained by the participants at baseline, at months 10 and month 20.

**Table 3:** SF-36 QoL scores of all the domains

SF-36 Norm-based scale	Items	Baseline (*N* = 46)		10th month (*N* = 32)		20th month (*N* = 28)		Alpha-Cronbach	*p*-value	
		Mean	SD	Mean	SD	Mean	SD			
Physical functioning (FP)	10	78.0	21.7	79.1	20.4	79.3	19.9	0.9	0.96	
Role Physical (RP)	4	47.8	50.5	46.1	46.3	63.4	43.8	1	0.3	
Role Emotional (RE)	3	58.0	43.6	63.5	44.3	69.1	39.5	0.9	0.55	
Vitality (VT)	4	57.3	25.6	61.7	21.5	65.4	19.5	0.8	0.33	
Emotional well-being (MH)	5	67.0	21.4	68.5	20.0	69.3	21.0	0.9	0.89	
Social functioning (SF)	2	69.0	25.9	77.3	21.9	79.5	23.9	0.8	0.14	
Pain (BP)	2	78.2	22.9	68.1	25.0	71.6	29.1	0.6	0.21	
General health (GH)	5	63.9	22.2	60.2	19.7	67.0	21.8	0.9	0.47	
8 item SF-36	35	64.9	32.3	65.6	30.5	70.6	28.8	N/A	N/A	
Mental health component summary (MCS)	14	62.8	30.6	67.8	29.0	70.8	27.3	N/A	0.33	
Physical health component summary (PCS)	21	67.0	33.9	63.3	31.9	70.3	30.3	N/A	0.53	

The difference in the PCS and MCS scores were compared stratified by sex, age groups and history of TB. The resulting p-values were non-significant stating that there were no differences in the MCS and PCS values based on sex and history of TB. There was a negative correlation between PCS score and the age group category “>/= 50” (p-value = 0.02) indicating that as the age increases, there is a decrease in the PCS score.

### Psychological health and well-being

**Fig. 5:**
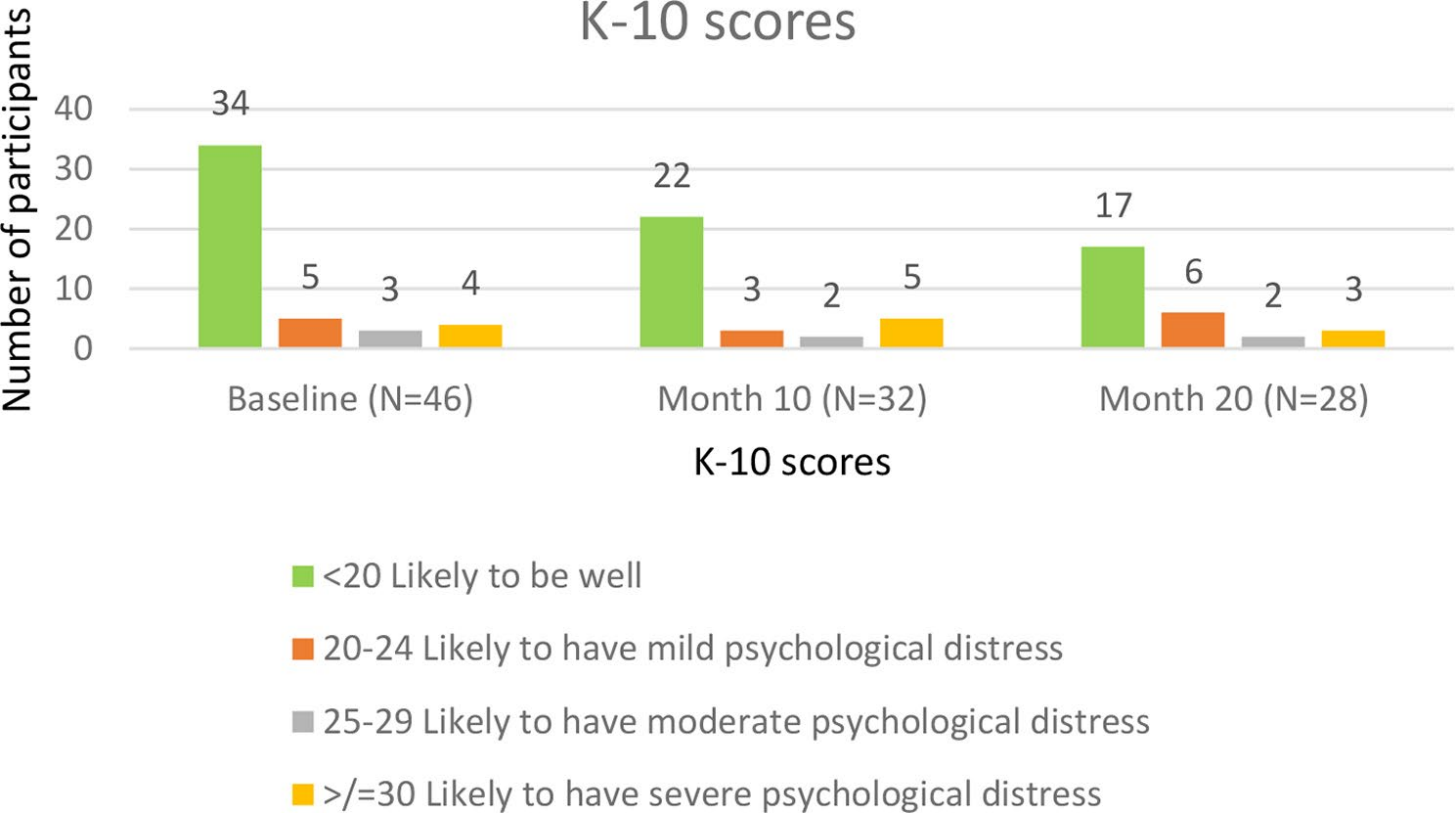
K-10 scores at baseline, month 10 and month 20

Majority of the participants (73.9%, 68.8%, 60,7%) reported a score less than 20 at baseline, month 10 and month 20, respectively. 3 (10.7%) of the participants had a score more than or equal to 30 at month 20, indicating severe psychological distress (Fig. 5). There was no difference in the scores during the treatment period (p-value = 0.78). The K-10 scores at month 20 were compared based on sex, age, history of TB, relationship status and employment status, and findings were non-significantly different.

## Discussion

We holistically examined the socio-economic impact, quality of life and psychological distress among patients affected by DR TB in Romania. In this study, before TB diagnosis, 19 (41.3%) of the participants had personal income more than the reported net salary in Romania. However, at month 20, only 5 (17.9%) had the income more than the average salary. Concerning quality of life, improvements in domains physical functioning, role emotional, vitality, emotional well-being, and social functioning were observed but the improvements were not significant enough. With reference to K-10 scoring, majority of the participants reported being mentally fit yet at month 20, 3 of the participants reported having severe psychological distress. The current study also identified common social practices and known risk factors, in particular the consumption of alcohol and smoking.

The participant’s demographic characteristics in the present study such as gender, age and education were similar to the Stoichita et al.’s study [5] in the same setting in Bucharest, Romania. Twenty-eight participants were employed in the formal sector of whom 25 were on paid sick leave at the baseline. Two received cash transfers meant for financially marginalized families, 1 received disability grant, and 1 received student allowance. Almost all the participants were insured except one who received medical allowance. Initially, a minor segment of the participants had sought financial assistance from friends or family members. By the month 20, this figure rose to 17.9%. Moreover, more than half (60.7%) of the participants received social welfare benefits, vouchers, or goods from the government. According to the Government Decision in 2018, which was officially implemented in January 2021, the monthly food allowance for patients treated on an outpatient basis is 16 RON/day (approximately 500 RON/month) [17]. Subsequently, this amount increased to approximately 900 RON/month according to another Government decision made in July 2022 [18]. This could be the reason for 50% of the patients to receive vouchers from the government. On the contrary, a study in the UK which determined the economic impact of TB, found that 3.0% of participants borrowed money to cover their medical expenses and 26.0% received financial support from family and friends. This response remained the same during the follow-up period [19]. Only 18% of them received social benefits [19].

During the treatment, personal income as well as the household income dwindled. In 2023, the minimum net salary in Romania was set at 1,863 RON (373.90€) [20]. Before receiving TB diagnosis, almost half of the patients had an income above the minimum salary. At month 20, less than one in 5 had an income above 1,800 RON. Several studies [21] have documented the expenses incurred by patients in the context of TB, encompassing catastrophic costs, expenses related to hospitalizationand other associated costs. Moreover, households with the lowest income face additional challenges as unexpected health expenditures on treatment contributing to a further decline in their economic well-being. Thus, it is imperative to implement interventions and strategies aimed at preventing catastrophic costs.

The HRQoL was measured using the SF-36 scale and there were no significant differences in the scores before and after the treatment. When age, gender and history of TB were compared with MCS and PCS values, no significant p-values were found except for a negative correlation between the age group “>/= 50 years” and PCS scores. Similar results were obtained in a study in Poland wherein there were no differences in the QoL scores between the sexes [22]. Another study in Nigeria also found that participants aged less than 50 had better QoL scores [23].

The psychological status of the participants was assessed using the Kessler scale questionnaire. At the baseline 26.1% of the participants and at the end of the 20th month almost 40% of participants had psychological distress of varying levels. Peltzer et al. [14] also found high rates of psychological distress among TB patients in South Africa. On the contrary, a study in Romania, wherein HADS scale was used to measure the prevalence of depression and anxiety among DR TB patients, found that the prevalence of depression and anxiety was as high as 46.0% and 43.0% at the baseline and was 50.0% and 39.0% at follow-up [5].

Romanian National Institute of Public health along with technical guidance from WHO released the National strategy for Tuberculosis control in Romania for the period of 2022 to 2030 [24]. This report lists the objectives of the National Tuberculosis Prevention, Surveillance and Control Program (NTPSCP) such as strengthening the health infrastructure, improving the access to diagnostic services, etc. and the ultimate goal of the program is to increase its efficiency, quality and accessibility, especially for people with disabilities, and for disadvantaged and isolated communities [24]. According to NTPSCP, the average cost for TB treatment with line I medication was 241 RON, and for DR TB case line II treatment was 3,446 RON [24]. Although the program lends financial support by bearing the cost of treatment for TB irrespective of the patient’s health insurance status, it doesn’t emphasise on mental health or psychological support for patients undergoing TB treatment [24]. Hence, a comprehensive approach for TB management is the need of the hour.

### Study limitations

The study has several limitations. Firstly, the study had a small sample size due to which the association between variables might not be detected. Secondly, since the study design was cohort study, patients withdrew from it at different time intervals and at the end month 20 only 28/46 (60.9%) participants responded to the questionnaires. Thirdly, the study didn’t calculate the direct and indirect costs associated with TB diagnosis and treatment, rather the change of income and coping mechanisms.

## Conclusion

The present study demonstrates the multifaceted impact of TB, not only on the quality of life but also on the economic situation and psychological well-being of the patients. In this study, personal and household income reduced drastically due to majority of the participants being on sick leave. While there was discernible improvement in the HRQoL and K-10 scores, the improvement was not statistically significant. In the future, research with a larger sample can produce more insightful results. Additionally, research on indirect and direct costs related to TB management needs to be conducted, which could identify hidden critical cost. Results of this study suggest that social and psychological support to the patients may ensure better standard of living during and post TB.

## Electronic Supplementary Material

Below is the link to the electronic supplementary material.

Supplementary Material 1

## Data Availability

The datasets used and/or analysed during the current study are available from the corresponding author on reasonable request.

## Abbreviations

ANOVA: Analysis of Variance
BMI: Body Mass Index
COPD: Chronic obstructive pulmonary disease
DZIF: German Centre for Infection Research
ERB: Ethics review boards
EU: European Union
HRQoL: Health-related quality of life
IRB: Institute review boards
K-10: Kessler psychological distress scale
DR TB: Drug resistant tuberculosis
MCS: Mental health component summary
NTPSCP: National Tuberculosis Prevention, Surveillance and Control Program
NTP: National Tuberculosis Program
PCS: Physical health component summary
RON: Romanian Leu
RR TB: Rifampicin-resistant tuberculosis
SF-36: Short Form-36 Health survey
STI: Sexually transmitted infections
TB: Tuberculosis
QoL: Quality of life
WHO: World Health Organization
XDR TB: Extensively drug-resistant tuberculosis
