# Supplementary material for: Impact of drug-resistant tuberculosis on socio-economic status, quality of life and psychological well-being of patients in Bucharest, Romania: a prospective cohort study
Source: J Health Popul Nutr. 2024 Dec 22;43:223. doi: 10.1186/s41043-024-00717-x (PMC11665218; doi:10.1186/s41043-024-00717-x)
Supplement: Supplementary file 1 — Supplementary Material 1 [file 41043_2024_717_MOESM1_ESM.docx]

Annex 1. **Comparison of characteristics between the participant group that completed the study and the group that discontinued from the study**

| Characteristics |  | Completed the study (N = 28) | Discontinued from the study (N = 18) | p-Value |
| --- | --- | --- | --- | --- |
| Age | 19-45 years | 13 | 6 | 1 |
|  | >45 years | 15 | 12 |  |
|  |  |  |  |  |
| Sex | Male | 20 | 13 | 0.58 |
|  | Female | 8 | 5 |  |
|  |  |  |  |  |
| Marital status | Single | 10 | 7 | 1 |
|  | Not Single | 18 | 11 |  |
|  |  |  |  |  |
| Education | Vocational training or till high school | 27 | 16 | 0.55 |
|  | University and above | 1 | 2 |  |
|  |  |  |  |  |
| Employment status | Employed | 8 | 9 | 0.29 |
|  | Unemployed | 19 | 9 |  |
|  |  |  |  |  |
| Previous history of TB | Yes | 12 | 9 | 0.86 |
|  | No | 16 | 9 |  |
